# DTIP-WINDGRU a novel drug-target interaction prediction with wind-enhanced gated recurrent unit

**DOI:** 10.1186/s12859-025-06141-0

**Published:** 2025-07-20

**Authors:** Kavipriya Gananathan, D. Manjula, Vijayan Sugumaran

**Affiliations:** 1 School of Computer Science Engineering (SCOPE), Vellore Institute of Technology, Chennai, 600127 India; 2 Department of Decision and Information Sciences, School of Business Administration, Oakland University, Rochester, MI 48309 USA; 3 Center for Data Science and Big Data Analytics, Oakland University, Rochester, MI 48309 USA

**Keywords:** Drug-target interaction, Prediction model, Deep learning, Gated recurrent unit, Wind driven optimization

## Abstract

**Background:**

Identification of drug target interactions (DTI) is an important part of the drug discovery process. Since prediction of DTI using laboratory tests is time consuming and laborious, automated tools using computational intelligence (CI) techniques become essential. The prediction of DTI is a challenging process due to the absence of known drug-target relationship and no experimentally verified negative samples. The datasets with limited or unbalanced data, do not perform well. The models that use heterogeneous networks, non-linear fusion techniques, and heuristic similarity selection may need a lot of computational power and experience to implement and fine-tune. The latest developments in machine learning (ML) and deep learning (DL) models can be employed for effective DTI prediction process.

**Results:**

To that end, this study develops a novel DTI Prediction model, namely, DTIP-WINDGRU Drug-Target Interaction Prediction with Wind-Enhanced GRU. The major aim is to determine the DTIs in both labelled and unlabelled samples accurately compared to traditional wet lab experiments. To accomplish this, the proposed DTIP-WINDGRU model primarily performs pre-processing and class labelling. In addition, drug-to-drug (D-D) and target-to-target (T-T) interactions are employed to initialize the weights of the GRU model and are employed for the, DTI prediction process. Finally, the Wind Driven Optimization (WDO) algorithm is utilized to optimally choose the hyperparameters involved in the GRU model.

**Conclusions:**

For ensuring the effectual prediction results of the DTIP-WINDGRU model, a widespread experimentation process was carried out using four datasets. This comprehensive comparative study highlighted the better performance of the DTIP-WINDGRU model over existing techniques.

## Introduction

In recent years, the prediction of drug-target binding affinity (DTA) has become increasingly pivotal in pharmaceutical research, facilitating the discovery and development of new therapeutic agents. Therefore, various computational methodologies have been developed to address this challenge, ranging from classical to advanced deep learning approaches.

The classical methods often relied on molecular docking simulations and quantitative structure–activity relationship (QSAR) models to predict DTA. These methods typically consider structural and chemical properties of both drugs and target proteins to estimate binding affinities. In contrast, novel approaches harness the power of machine learning and artificial intelligence, particularly deep learning. For instance, DeepDTA introduced by Öztürk et al. leverages deep neural networks to predict binding affinities by learning from large-scale datasets [1], while,WideDTA, proposed by Öztürk et al., expands this by utilizing wide and deep learning architectures to capture both local and global interactions [2].

Graph neural networks (GNNs) have also gained prominence in DTA prediction, exemplified by GraphDTA, which integrates molecular graphs to model complex interactions between drugs and targets [3]. MSGNN-DTA further enhances predictive accuracy by fusing multi-scale topological features using GNNs [4]. He et al., integrated sequence and graph information, which further improved prediction capabilities [5]. Moreover, advancements like BindingSite-AugmentedDTA [6] and NHGNN-DTA highlight the trend towards interpretable models that elucidate the molecular basis of binding affinity predictions, crucial for drug repurposing and personalized medicine [7].

It is important to note that predicting those interactions is a pre-requisite for other processes namely drug-drug interactions prediction, drug repurposing, and side effect prediction [8]. The procedure of DTI prediction takes into account the various effects of the drug on protein named targets [9]. In this context, there are some popular DTIs that have a greater effect on accuracy. The process of drug development entails disease analysis, followed by discovery and development, preclinical research, clinical research, FDA reviews, and FDA post market safety monitoring. This procedure is also time-consuming, expensive, and laborious. Drug approval takes time since it needs to undergo rigorous laboratory testing. However, years of testing, may come up short since the medicine may only have minor side effects and not have a major impact on a person's health or well-being. In order to effectively treat an illness, a newly produced medication must be cost-effective, easy to get, and suitable for every individual. However, many medications never reach the market because they are too expensive or may have too many negative effects. Due to the fact that wet lab procedures utilized to make medications for novel diseases take time and may fail in the final clinical trial stage, innovative approaches are now being tried.

Discovering DTI in the lab is expensive and time consuming, and also, the presence of human error is an open challenge in this process. However, computation based DTI prediction is a reliable, inexpensive, viable alternate to blinded laboratory method [10]. It is essential to note that prediction of DTI becomes a link prediction challenge. As a starting point, prediction of DTI faces the problem of a heterogeneous network that could not be resolved by the link prediction method. The network might have two data structures: single-relational involving single type of link or multi-relational including multiple types of links. The computational DTI prediction method is categorized into two types: network-based, and machine learning-based models. The machine learning (ML) based method combines known interaction, drug information, and target information [11] where this information is fed to the learning algorithm. Target domain information and Drug domain information is converted into feature vectors or similarity matrices and the conventional method treats the non-interaction information as negative samples that are not reasonable as non-interaction information, which might have undetected drug-target interaction [12]. Many of the existing techniques are incapable of predicting new drugs without any targets that actually limit the application [13].

Predicting DTIs is a complex task requiring integration of biological data, machine learning, and domain expertise. Feng et al. [14] proposed a deep learning model that significantly improves DTI prediction by using Position Specific Scoring Matrix (PSSM) and Legendre Moment (LM) to extract evolutionary protein features. This model combines protein features with drug molecular substructure fingerprints to create informative feature vectors for drug-target pairs. The Deep Long Short-Term Memory (DeepLSTM) networks are employed to handle sequential data, addressing temporal aspects of drug-target interactions effectively, even with small datasets, while Sparse Principal Component Analysis (SPCA) is used to fuse PubChem fingerprint data with protein evolutionary features, enhancing feature expression and potentially improving model accuracy. Öztürk et al. [1] introduced DeepDTA, a deep learning approach to predict drug-target binding affinities using a convolutional neural network to process drug and protein sequences, but its high computational costs and potential overfitting due to the large number of parameters involved is a potential drawback.

Similarly, Wang et al. [16] presented an automated approach for predicting DTIs, reducing the time and cost of traditional experimental methods. This method leverages deep learning and stacked autoencoders to extract representative features from protein sequences and drug chemical structures combining information on drug molecular structure and protein amino acid sequence, recognizing their combined impact on drug-target connections. The experimental results demonstrate superiority over alternative feature extraction algorithms, classifiers, and current methods in terms of performance. However, the drawbacks lie in the limited interpretability of deep learning models and dependency on the availability and quality of biological data.

DDR (Drug-target interaction prediction) by Olayan et al. [15] uses a heterogeneous network that incorporates diverse drug-drug and protein–protein similarities, alongside established DTIs, employing graph mining algorithms. It integrates various drug-drug and protein–protein similarities through a non-linear fusion approach, enhancing prediction accuracy, capturing complex and non-linear interactions. This method employs heuristic similarity selection in preprocessing to focus on relevant similarities, reducing noise and improving prediction quality. DDR's flexibility in handling diverse prediction tasks, parameters, datasets, and evaluation methods underscores its utility in DTI prediction. The work by Beck et al. [17] focused on repurposing approved medications for SARS-CoV-2 treatment, expediting drug development due to their prior safety evaluations. The deep learning-based model (MT-DTI) excels in capturing intricate connections between medications and viral proteins without heavy reliance on domain-specific information. It identifies pharmaceuticals interacting with SARS-CoV-2 viral proteins, contributing valuable insights into COVID-19 therapeutic options. AutoDock Vina, a traditional 3D structure-based model, highlights MT-DTI's strengths and differences, emphasizing its advantages. The study expands the search to include medications for asthma, COPD, and immunosuppression, unveiling additional therapeutic options. It ranks medication candidates based on binding affinities to viral proteins, guiding further research. However, computational demands for handling heterogeneous networks and sensitivity to hyperparameter and similarity measure choices pose challenges. Furthermore, the study's focus on 3D structure-based models limits comparisons with contemporary methods, potentially overlooking alternative approaches. The MT-DTI model may not entirely avoid domain-specific information and the limited Comparative Analysis study provides a comparison with a 3D structure-based model lacks comparison with other contemporary methods.

A variety of aspects, including sequence-based, structure-based, fingerprint-based, and evolution-based data for describing drug and target information were used by Chen et al. [19]. This method of fusing many pieces of information improves how medication and target pairings are represented. The best features were found without compromising prediction accuracy by using XGBoost for feature selection which enhances the effectiveness and interpretability of the model. A deep neural network with a layer-by-layer learning method is used in the DNN-DTIs model. This deep learning architecture is appropriate for DTI prediction problems because it can extract high-level characteristics from raw DTI data. Thus, it can be concluded that DNN-DTIs is a reliable predictor for DTIs. DNN-DTIs are well suited for a wide range of applications, including drug repositioning, drug design, and understanding drug-target interactions, all of which have major implications for drug discovery because of their precise prediction performance. The major constraint lies in the potential difficulties associated with hyperparameter tuning for achieving optimal performance. Additionally, the approach is hindered by a restricted exploration of advanced deep learning architecture.

The key benefit of the model developed by Zhang et al. [20] emphasizes the acquisition of molecular structure data for medicinal molecules, crucial for understanding DTIs. The model leverages transformer networks, known for their strong performance in natural language processing and sequence-based tasks, effectively capturing interactions and long-range dependencies within chemical structures. It integrates multilayer graph information, revealing intricate patterns unobservable with traditional methods. Additionally, the incorporation of a convolutional neural network (CNN) extracts local residue information from target sequences, enhancing prediction accuracy and the model was tested on DrugBank dataset to demonstrate superior performance compared to models relying solely on target sequence data, highlighting the significant impact of molecular structural information on DTI prediction. The model's potential for drug repositioning is evident in its ability to identify therapeutic medications for diseases like COVID-19 and Alzheimer's, accelerating the discovery of new applications for existing drugs. This can hasten the identification of fresh uses for already-available medications. However, handling extensive datasets may necessitate significant computational resources. The GraphDTA model proposed by Nguyen et al. [3] utilizes a graph neural network to predict drug-target binding affinity by capturing the complex relationships between molecular structures and biological targets. It mainly emphasizes the feature extraction techniques to improve DTI prediction accuracy, but the model’s performance also heavily relies on the quality of the graph representation, which may not effectively capture all relevant features for drugs and targets when dealing with complex chemical structures.

Wang et al. [21] use CNN for predicting DTIs that can be organized using a knowledge graph that is created from drug-target combinations utilizing current information in an organized way which is the goal of this strategy. In order, to extract complicated information from drug-target pairings, a Convolutional-Convolutional (Conv-Conv) module is used, followed by a fully connected neural network, which can improve prediction accuracy. The use of Knowledge Graph based DTI (KG-DTI) to reposition medications for Alzheimer's disease (AD) by targeting apolipoprotein E shows potential and the top-recommended medications gives experimental proof that it may be repositioned to treat AD. Zhao et al. [18] proposed method Graph Convolutional network (GCN-DTI) main benefit is the associations between Drug-Protein Pairs (DPPs) into DTI modeling. GCN enables the extraction of features from DPPs, capturing intricate linkages and network interactions. Here, numerous graph-based tasks have been successfully completed by GCNs. The capacity of the model to identify between real and fake DPP characteristics is increased when GCN and a DNN are used for prediction. GCN-DTI performs noticeably better than state-of-the-art techniques. This demonstrates how well it can identify DTIs. Additionally, there is a shortage of discussion regarding the management of noisy or incomplete information within the graph.

NHGNN-DTA, a node-adaptive hybrid graph neural network proposed by He et al. [7] predicts the binding affinity between drugs and targets. It offers enhanced interpretability and accuracy, but the selection of node features and hyperparameters, is a tedious task which can impact the overall performance further, the complexity of the hybrid graph neural network may require significant computational resources and time for training and tuning.

The challenges faced by using unbalanced data in DTI prediction was solved by Rajpura et al. [22], using a Positive Unlabeled (PU) learning strategy and a one-class support vector machine (SVM) instead of marking unknown pairings as negative. This method considers them as unlabeled, which can improve prediction accuracy through an assessment of drug features thanks to the integration of 4860 Klekota Roth fingerprints and 881 PubChem fingerprints as high-dimensional feature representations for medicines. A thorough knowledge of the suggested approach's performance in diverse circumstances is provided by the evaluation of it using multiple formulations, including known drug-known target, known drug-unknown target, unknown drug-known target, and unknown drug-unknown target settings. It is important to note that by eliminating the presumption that all unknown interactions are negative, Lan et al.’s [23] use of positive-unlabeled learning in the PUDT technique successfully overcomes the problem of dealing with unknown interactions. The integration of several biological data sources pertaining to target proteins by PUDT can result in a more comprehensive understanding of drug-target interactions. This may increase prediction accuracy by capturing a wider range of target protein characteristics. PUDT takes target protein structure into account in addition to sequence information. This technique proves the capacity to find possible drug-target interactions, which is useful for drug discovery and design, by reviewing the literature and data bases that are currently accessible while, Chen et al. [8] reviewed various machine learning approaches for DTI prediction, including Positive-Unlabeled learning strategies which struggles with noisy or incomplete data thereby resulting in inaccurate prediction.

Haddadi et al.’s [24] positive-unlabeled learning (PUL) technique broadens BLM to improve the predictability of drug-target interactions. It offers a more accurate depiction of the dataset by treating all unlabeled interactions as unlabeled. Drug development is made more productive by using computational techniques like BLM with PUL instead of labour-intensive laboratory studies to anticipate drug-target interactions. These approaches face certain constraints including sensitivity to the selection of fingerprints and feature representations with a restricted usage of advanced machine learning techniques. However, the methods depend on the availability and reliability of diverse biological data sources, with limited investigation into interactions beyond enzymes, ion channels, GPCRs, and nuclear receptors. They also rely on the assumption that unlabeled interactions are genuinely negative and face challenges with noisy or insufficient data.

The earlier studies mentioned in the literature had a number of drawbacks. Although, the models used in the literature perform well with limited datasets, it's crucial to take into account whether the findings can be applied to a wider variety of drug-target interactions. The datasets with limited or unbalanced data, could not perform well. The models that use heterogeneous networks, non-linear fusion techniques, and heuristic similarity selection may need a lot of computer power and experience to implement and fine-tune. The choice of hyperparameters and similarity measures may have an impact on how well these approaches work, and adequate parameter tweaking, take time to get the best results. The presented models have a high degree of accuracy, but there is still opportunity for development and research methods such as gated recurrent units and recurrent neural networks (RNN), which would improve DTI prediction and data mining. The predictions were impacted by noisy or insufficient data sources, in order, to overcome the lack of confirmed negative samples and increase prediction accuracy, positive-unlabeled learning is used, however its effectiveness may vary depending on the dataset's properties and parameter selections. Docking techniques favour interactions for whose structural details are known while excluding fresh DTIs with unidentified structures. In conclusion, the approaches stated in the literature does not addresses all the difficulties associated with predicting drug-target interactions, but our proposed method deals with significant difficulties in predicting DTIs.

Our proposed novel Drug-target interaction prediction with Wind-Enhanced GRU (DTIP-WINDGRU) model’s pre-processing and class labeling are the main functions of DTIP that we have suggested. Additionally, D-D and T-T interactions are used for DTI prediction as well as to initialize the weights of the GRU model. The inclusion of D-D and T-T interactions in initializing the weights of the GRU model implies an effort to capture recurring patterns and enhances the generality of the model. The inclusion of GRU indicates an effort to investigate more cutting-edge methods for DTI prediction. In order to choose the GRU model's hyperparameters optimally, the WDO technique is used. The WDO algorithm's use for hyperparameter optimization implies an effort to automatically choose complicated model parameters. As a result, researchers without extensive experience in hyperparameter tuning may find the model easier to use. The WDO technique may be used to pick hyperparameters and aid in quickly locating the best ones which paves way for the questions raised about the selection of hyperparameters. The WDO approach to optimize the model may assist reduce problems caused by noisy or inadequate data. GRU and WDO indicates an alternate method to DTI prediction that may get around some of the problems with docking approaches, even if the precise limits of docking techniques are not highlighted in the literature. In order to ensure the DTIP-WINDGRU model's predictive outcomes are accurate experimental approach is carried out on four datasets.

The remainder of this paper is organized as follows. Sect. “Methods” explains the methods proposed and Sect. “Results and discussion” provides a detailed description of the results and discussion and Sect. “Conclusion” concludes the paper.

## Methods

### The proposed model

**Fig. 1:**
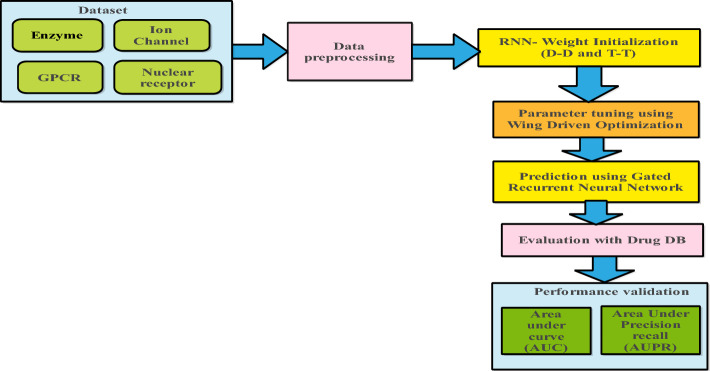
Process Model for the DTIP-WINDGRU Approach

The DTIP-WINDGRU model uses pre-processing procedures to get the data ready for prediction. Additionally, class labeling is involved, which entails giving known interactions labels (such as positive or negative) for unlabelled instances in the data. D-D and T-T interactions are taken into consideration during the actual prediction process using the GRU. On the account that the protein sequences are lengthy, a basic CNN is unable to adequately capture the context dependencies within them. Consequently, we enhance it by interpreting the protein sequences as time series and use the GRU to extract their features. In order to ensure that the model performs better in protein feature extraction, we employ GRU to capture the long-term dependencies in order to handle the feature extraction problem for lengthy amino acid sequences. The findings of the four models have been improved to varying degrees. A GRU, a kind of recurrent neural network (RNN), is at the centre of the model. The model's weights are initialized using these interactions, facilitating the model's ability to learn and produce accurate predictions. The model uses the WDO method in the concluding step. The GRU model's optimal hyperparameters are chosen using the model behavior-influencing settings, which is essential for obtaining reliable findings. A new method for DTI prediction combines a GRU model with D-D and T-T interactions. It is potentially beneficial to optimize hyperparameters using the WDO technique. The model's versatility in tackling DTI prediction difficulties is indicated by its ability to handle labeled and unlabeled data. Overall, the proposed DTIP-WINDGRU model provides a unique viewpoint on DTI prediction and includes innovative components to enhance precision and effectiveness in this significant field of study. The process model for the DTIP-WINDGRU approach is shown in Fig. 1.

The step-by-step explanation of the model’s workflow includes:

**Step 1: Data Pre-processing:**

**Class Labeling:** We assign labels to known interactions (positive or negative) for previously unlabeled instances in the dataset.

**Interaction Consideration:** The D-D and T-T interactions are taken into account to enrich the data.

**Step 2: Feature Extraction:**

**Protein Sequence Processing:** The protein sequences are interpreted as time series data.

**GRU:** A GRU is employed to extract features, capturing long-term dependencies within the sequences.

**Step 3: Model Initialization:**

**Weight Initialization:** The model's weights are initialized using the interaction data, facilitating effective learning.

**Step 4: Prediction Process:**

**GRU-Based Prediction:** The GRU model processes the input data to predict drug-target interactions.

**Step 5: Hyperparameter Optimization:**

**WDO Method:** The WDO technique is used to optimize the GRU model’s hyperparameters, ensuring reliable and accurate predictions.

### Dataset description

The performance of the proposed method is validated by utilizing the gold standard dataset. The databases DrugBank, KEGG BRITE, SuperTarget, BRENDA, and others are used to find drug-target interactions but due to bias concerns caused by the greater number of negative samples than positive data, DTI pairings only include positive interactions since negative interactions have not been empirically proven. The negative interactions must be reprocessed to return to their original state since they lack experimental support, which may result in the emergence of new positive interactions.

The gold standard dataset was originally compiled by Yamanishi et al. (2008), integrating information from multiple databases including DrugBank, BRENDA, SuperTarget, and KEGG BRITE. It provides benchmark datasets for four classes of target proteins: Enzymes, Ion Channels, G-Protein Coupled Receptors (GPCRs), and Nuclear Receptors. Each dataset consists of known positive drug-target interactions, with drugs and targets represented using chemical structure and protein sequence similarity matrices respectively. The dataset is publicly available at: http://web.kuicr.kyoto-u.ac.jp/supp/yoshi/drugtarget/

### Pre-processing and class labeling

In the proposed work, we have used four DTIP data sets such as Ion Channel, Enzyme, Nuclear Receptor, and GPCR. The drug chemical data is attained in DRUG AND COMPOUND Section in the KEGG LIGAND. The chemical formation resemblance amongst the components is defined by SIMCOMP which provides a scoring value based on the size of substructure with graph alignment. The target protein sequence similarity is evaluated using the Smith-Waterman technique. It is used to achieve local sequence alignment and to find similarities between two sequences (target protein amino acid sequences). The Smith-Waterman algorithm concentrates on locating the best local alignments, useful for locating homologous or functional domains in proteins by understanding the connections and probable functional similarity between various proteins. The sequence similarity amongst the target is defined by the normalized Smith–Waterman method, based on the data of amino acid order of target protein derived from KEGG GENE data base. A pair of proteins $A_{i}$ and $A_{j}$, the sequence similarity amongst them can be defined by the following equation:

1 $$Sim_{{seq\left( {A_{i} ,A_{j} } \right)}} = \frac{{SW\left( {A_{i} ,A_{j} } \right)}}{{\sqrt {SW\left( {A_{i} ,A_{i} } \right)} \sqrt {SW\left( {A_{j} ,A_{j} } \right)} }}$$

where $SW\left( {A_{i} , A_{j} } \right)$ denotes the score of Smith–Waterman method. Next, the proposed approach undergoes class labelling where the target interaction exists in the dataset are utilized for labelling the unknown instance.

The class labeling method categorizes instances in a dataset, particularly those connected to target interactions. The process of class labeling entails categorizing or labeling the data instances using target interactions. The target interactions and their weights are used to label the unlabeled instances in the DTI dataset. The unknown instances, or data points without labels indicating whether the interaction exists or not, are subjected to the class labelling procedure. This technique labels ambiguous occurrences by using the positive interactions from known target interaction information. The unknown instances are classified as positive interactions if they have similarities with known target interactions; otherwise, they are classified as negative interactions. In essence, this class labeling phase uses weights from RNN, where the algorithm learns from labeled examples to generate predictions or classifications on fresh, unlabeled data, to help categorize data instances in the dataset.

### Design of GRU based predictive model

**Fig. 2:**
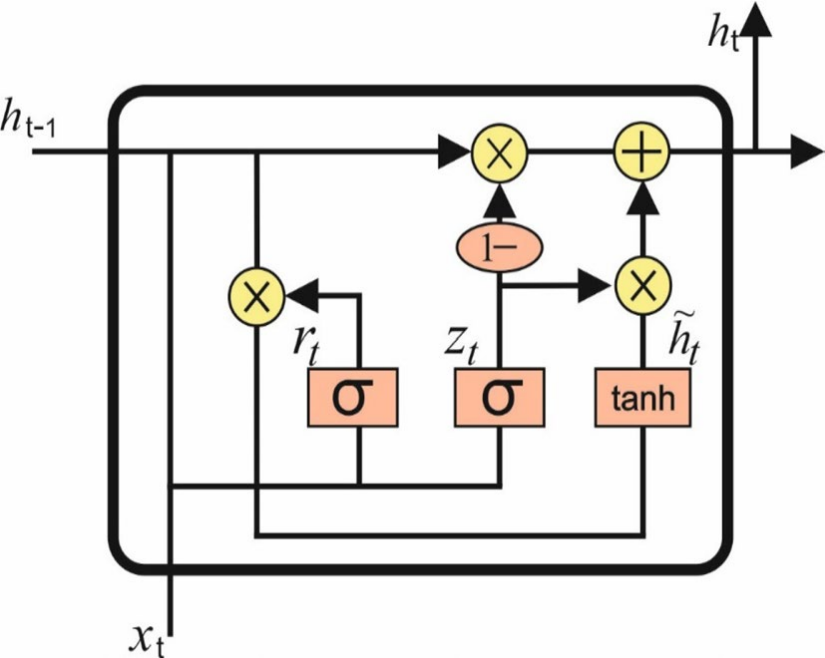
Framework of GRU

In a RNN, the NN utilizes recurrent connection in all the units. The activations of neurons are fed into weights, and each unit incorporates a time delay along with a hidden memory value. This enables the network to learn the temporal dynamics of successive information by retaining historical activations [22]. Assuming a temporal input sequence $a^{l} = \left( {a_{1}^{l} ,{ } \ldots ,a_{T}^{l} } \right)$ of length $T$ ($a_{t,i}^{l}$ the activation of unit $i$ in hidden layer $l$ at time $t$), an RNN maps to a series of hidden value $h^{l} = \left( {h_{1}^{l} ,{ } \ldots ,h_{T}^{l} } \right)$ and outputs a sequence of activation $a^{{\left( {l + 1} \right)}} = \left( {a_{1}^{{\left( {l + 1} \right)}} ,{ } \ldots ,a_{T}^{{\left( {l + 1} \right)}} } \right)$ by iterating through the following equation:

2 $$h_{t}^{l} = \sigma \left( {W_{xh}^{l} a_{t}^{l} + h_{t - 1}^{l} W_{hh}^{l} + b_{h}^{l} } \right)$$

where $\sigma$ refers the nonlinear activation function, $b_{h}^{l}$ signifies the hidden bias vector and $W$ stands for the weight matrices, $W_{xh}^{l}$ represents the input‐hidden weight matrix and $W_{hh}^{l}$ implies the hidden‐hidden weight matrix. It represents the weights connecting the hidden layer at time t − 1 to the hidden layer at time t in the RNN equation [9]. Here, $W_{hh}^{l}$ captures the influence of the previous hidden state on the current hidden state. It is a matrix of weights governing the recurrence in the hidden layer. It was determined in the following:

3 $$a_{t}^{{\left( {l + 1} \right)}} = h_{t}^{l} W_{ha}^{l} + b_{a}^{l}$$

where $W_{ha}^{l}$ denotes the hidden activation weight matrix and $b_{a}^{l}$ signifies the activation bias vector. It is worth highlighting the weight matrix $W^{l}$ determined for the MLP corresponds to $W_{xh}^{l}$ matrix in Eq. (2). Figure 2 depicts the structure of GRU.

These kinds of networks have Turing abilities [23] and, therefore, they are suitable for learning sequences but the memory model makes learning difficult to handle real‐time sequence processing. The LSTM extended RNN with memory cell, rather than recurrent unit, for storing and resultant data, easing the learning of temporal connections on long time scale. The GRU has a basic form of LSTM that is also a type of RNN. Unlike LSTM, GRU combines forget and input gates. In consideration of a network with h hidden layers, a small‐batch input at a given time step of $t$ is denoted as $X_{t} \in {\mathbb{R}}^{{n{*}d}}$, and the hidden state at the preceding time step t1 is $H_{t - 1} \in {\mathbb{R}}^{{n{*}h}}$. The resulting hidden layer $h$ of single GRU at existing time step $t$:

4 $$R_{t} = \sigma \left( {X_{t} W_{xr} + H_{t - 1} W_{hr} + b_{r} } \right)$$

5 $$Z_{t} = \sigma \left( {X_{t} W_{xz} + H_{t - 1} W_{hz} + b_{z} } \right)$$

6 $$\overline{H} = {\text{ tan }}h\left( {X_{t} W_{xh} + \left( {R_{t} E \odot H_{t - 1} } \right)W_{hh} + b_{h} } \right)$$

7 $$H_{t} = \left( {1 - Z_{t} } \right) \odot H_{t - 1} + Z_{t} E \odot \tilde{H}_{t}$$

Here, $\sigma$ represents the sigmoid activation function, defined as $\sigma \left( x \right) = 1/1 + e^{ - x}$. The weights connecting the hidden layer and update gate are represented by $W_{hz}$. The weights ${\text{connecting input layer and reset gate are denoted by }}W_{xr} , while W_{hz} {\text{and }}W_{xr}$ represents the hidden layer and reset gate, input layer and update gate. Here, $b_{r}$ and $b_{z}$ indicate the bias of reset and update gates, while $H_{t}$ points toward the candidate hiding state of existing time step denoted by $t.{ }$ The symbol $\odot$ represent the matrix multiplication of two components, and $Tanh$ characterizes the hyperbolic tangent activation function:

8 $${\text{ tanh }}\left( x \right) = 1 - \frac{2}{{1 + e^{ - 2x} }}$$

### Hyperparameter optimization

The optimal adjustment of the hyperparameters involved in the GRU model, includes WDO algorithm. The WDO algorithm is derived from the concept of atmosphere driven by forces such as wind, exhibit optimization-like characteristics. It is believed that by integrating these ideas into an optimization algorithm, the WDO method provides special benefits in terms of effectiveness and scalability [24]. The potential utility of the WDO algorithm lies in its capacity to navigate complex solution spaces and identify optimal configurations [31]. The algorithm has characteristics advantageous for maximizing the selection of therapeutic targets inspired by atmospheric dynamics. It is used to improve prediction accuracy with specific biological targets, contributing to the drug discovery process by precisely identifying potential therapies. The basis of WDO approach is Newton’s second law of motion that is utilized for providing precise results for analyzing atmospheric motion in the Lagrangian [24]:

9 $$\rho \vec{\alpha } = \sum \vec{F}_{i} ,$$

where $\vec{\alpha }$ represent the acceleration, $\rho$ indicates the air density for infinitesimal air parcel, and $\vec{F}$ denotes each force act on the air parcel. The air pressure establishes the relation with air parcel temperature and density, it can be shown as follows

10 $$P = \rho RT,$$

Now $P$ indicates the pressure, $R$ denotes the universal gas constant, and $T$ represents the temperature. The reason of air movement is because of the integration of several forces, largely including gravitational force $\left( {\vec{F}_{G} } \right)$, pressure gradient force $\left( {\vec{F}_{PG} } \right)$, Coriolis force $\left( {\vec{F}_{C} } \right)$, and friction force $\left( {\vec{F}_{F} } \right)$. it is described in the following

$$\vec{F}_{G} = \rho \delta V\vec{g},$$

11 $$\vec{F}_{PG} = - \nabla P\delta V,$$

$$\vec{F}_{C} = - 2\Omega \times \vec{u},$$

$$\vec{F}_{F} = - \rho \alpha \vec{u},$$

where $\delta V$ denotes finite volume of the air, $\vec{g}$ indicates the gravitational acceleration, $\nabla P$ characterizes the pressure gradient, $\Omega$ denotes rotation of the Earth, $\vec{u}$ epitomizes the velocity vector of the wind, and $\alpha$ signifies the friction coefficient. The abovementioned forces are added to (10).

12 $$\rho \frac{{\vartriangle \vec{u}}}{\vartriangle t} = \left( {\rho \delta V\vec{g}} \right) + \left( { - \nabla P\delta V} \right) + \left( { - 2\Omega \times \vec{u}} \right) + \left( { - \rho \alpha \vec{u}} \right),$$

where the acceleration $\vec{\alpha }$ in (1) is expressed as $\vec{\alpha } = \vartriangle \vec{u}/\vartriangle t$; for simplicity set $\vartriangle t = 1$; for infinitesimal air parcel, set $\delta V = 1$, that simplify (4) to

13 $$\rho \vartriangle \vec{u} = \left( {\rho \vec{g}} \right) + \left( { - \nabla P} \right) + \left( { - 2\Omega \times \vec{u}} \right) + \left( { - \rho \alpha \vec{u}} \right).$$

Based on (11), the density $\rho$ is expressed interms of the pressure;

14 $$\vartriangle \vec{u} = \vec{g} + \left( { - \nabla P\frac{RT}{{P_{cur} }}} \right) + \left( {\frac{{ - 2\Omega \times \vec{u}RT}}{{P_{cur} }}} \right) + \left( { - \alpha \vec{u}} \right) ,$$

where $P_{cur}$ indicates the pressure of present position. It is considered in the WDO approach [32] that position and velocity of the air parcel change at all the iterations. Therefore, $\vartriangle \vec{u}$ is expressed as $\Delta \vec{u} = \vec{u}_{new} - \vec{u}_{cur}$, in which $\vec{u}$ indicates the velocity in following iteration and $\vec{u}$ indicates the velocity at the present iteration. $\vec{g}$ and $\nabla P$ denotes vectors, they are broken down in direction and magnitude as $\vec{g} = \left| g \right|\left( {0 - x_{cur} } \right),$ $- \nabla P = \left| {P_{opt} - P_{cur} } \right|\left( {x_{opt} - x_{cur} } \right),$ $P_{opt}$ denotes the optimal pressure point, $x_{opt}$ indicates the optimal position, and $x_{cur}$ represent the existing position; update (14) with the new equation, (14) as follows

15 $$\vec{u}_{new} = \left( {1 - \alpha } \right)\vec{u}_{cur} - gx_{cur} + \left( {\frac{RT}{{P_{cur} }}\left| {P_{opt} - P_{cur} } \right|\left( {x_{opt} - x_{cur} } \right)} \right) + \left( {\frac{{ - 2\Omega \times \vec{u}RT}}{{P_{cur} }}} \right).$$

At last, there are three further substitutions required. The actual pressure value is substituted with rank amongst each air parcel according to the pressure value, the resultant formula of updating the velocity is defined in the following [19]:

16 $$\vec{u}_{new} = \left( {1 - \alpha } \right)\vec{u}_{cur} - gx_{cur} + \left( {RT\left| {1 - \frac{1}{i}} \right|\left( {x_{opt} - x_{cur} } \right)} \right) + \left( {\frac{{c\vec{u}_{cur}^{other dim} }}{i}} \right)$$

17 $$\vec{x}_{new} = \vec{x}_{cur} + \left( {\vec{u}_{new} \times \Delta t} \right),$$

whereas $i$ denotes the ranking amongst each air parcel and $\vec{x}_{new}$ indicates the novel position for the following iteration.

The WDO system develops a fitness function (FF) for reaching enhanced classifier results. It defines a positive integer for demonstrating the best efficiency of the candidate solution. In case of, minimizing of the classification error rate was regarded as FF is offered in Eq. (18).

$$fitness\left( {x_{i} } \right) = Error\,Rate\left( {x_{i} } \right)$$

18 $$= \frac{number\; of\; misclassified\; samples}{{Total \;number\; of\; samples}}*100$$

In the earlier sections, a prediction model based on the GRU with wind-driven optimization techniques [25] to carry out a thorough hyperparameter optimization was designed to predict DTIs. The complex process of parameter tuning plays a vital link between the design of our proposed approach and the validation stage that follows. As we proceed with the validation phase, our aim is to provide a smooth transition between these stages described in Fig. 1 and clarify how the improved parameters and the model’s approach supports the stability and effectiveness of the prediction model.

## Results and discussion

In this section, the performance validation of the DTIP-WINDGRU model is carried out using four datasets [26] namely enzyme, Ion channel, GPCR, and nuclear receptor datasets. The details related to the dataset are given in Table 1.

**Table 1:** Dataset Descriptions

Database	Drug	Target	Interactions
Enzyme-dataset	445	664	2926
Ion channel-dataset	210	204	1467
GPCR-dataset	223	95	635
Nuclear receptor-dataset	54	26	90

**Fig. 3:**
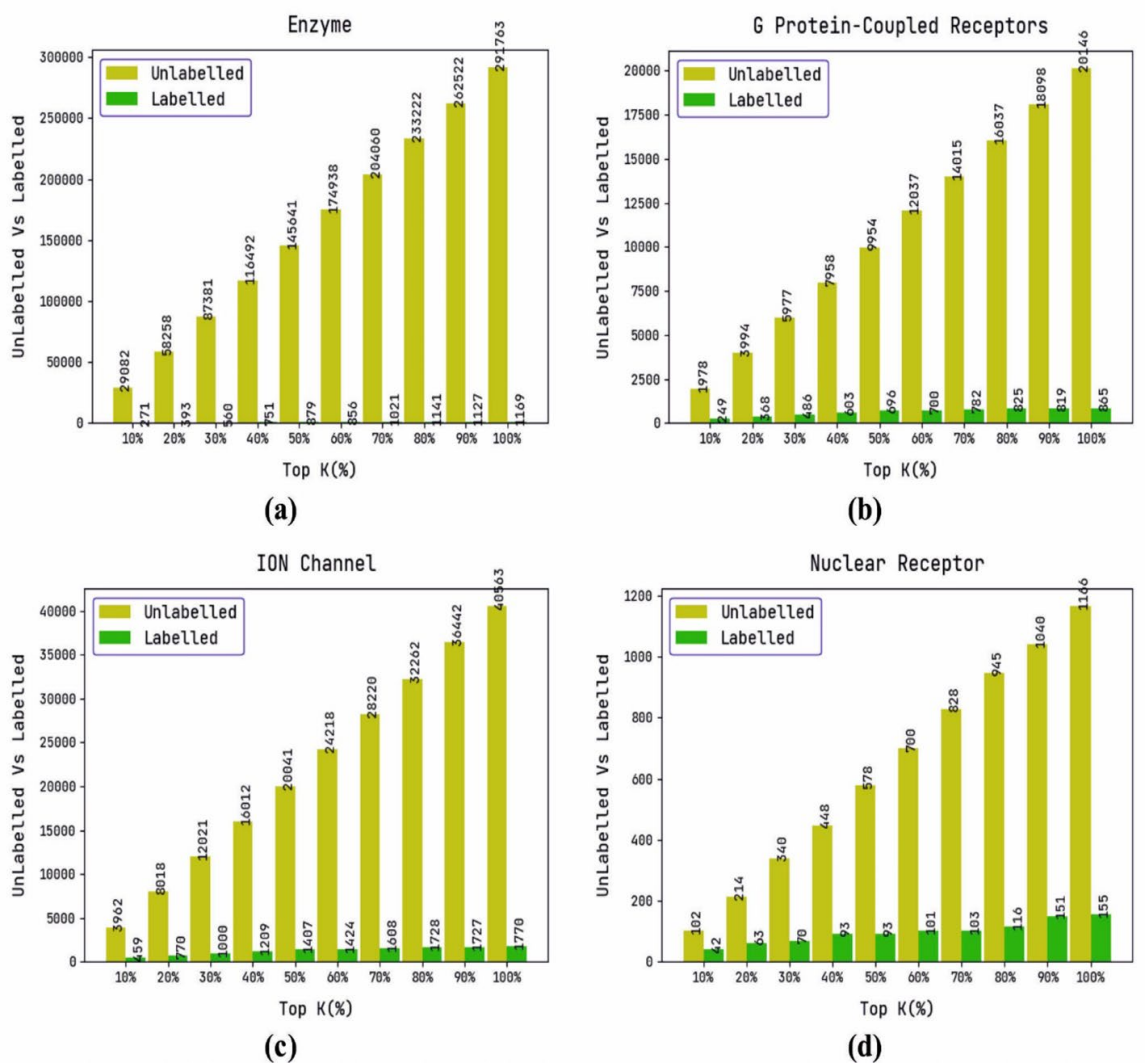
Labeled Data Analysis of DTIP-WINDGRU model. **a** Enzyme dataset, **b** G Protein-Coupled Receptors dataset, **c** ION Channel dataset, **d** Nuclear Receptor dataset, showing labeled and unlabeled instances at various top k% values

Table 2 and Fig. 3 portray the labeled and unlabeled results offered by the DTIP-WINDGRU model on distinct top $k$ values. The enzyme dataset with $k = 10\%$, DTIP-WINDGRU model has obtained 29,082 and 271 unlabeled and labeled instances respectively. In addition, on GPCR dataset with $k = 10\%$, the DTIP-WINDGRU model has attained 1978 and 249 unlabeled and labeled instances respectively. Simultaneously, on ION channel dataset with $k = 10\%$, the DTIP-WINDGRU model has gained 3962 and 459 unlabeled and labeled instances respectively while on nuclear receptor dataset with $k = 10\%$, the DTIP-WINDGRU model has provided 102 and 42 unlabeled and labeled instances respectively.

**Table 2:** Labeled data analysis of DTIP-WINDGRU model

Enzyme dataset			GPCR dataset		
Top k (%)	Unlabelled	Labelled	Top k (%)	Unlabelled	Labelled
10	29,082	271	10	1978	249
20	58,258	393	20	3994	368
30	87,381	560	30	5977	486
40	116,492	751	40	7958	603
50	145,641	879	50	9954	696
60	174,938	856	60	12,037	700
70	204,060	1021	70	14,015	782
80	233,222	1141	80	16,037	825
90	262,522	1127	90	18,098	819
100	291,763	1169	100	20,146	865
*ION channel dataset*			*Nuclear receptor dataset*		
10	3962	459	10	102	42
20	8018	770	20	214	63
30	12,021	1000	30	340	70
40	16,012	1209	40	448	93
50	20,041	1407	50	578	93
60	24,218	1424	60	700	101
70	28,220	1608	70	828	103
80	32,262	1728	80	945	116
90	36,442	1727	90	1040	151
100	40,563	1770	100	1166	155

**Fig. 4:**
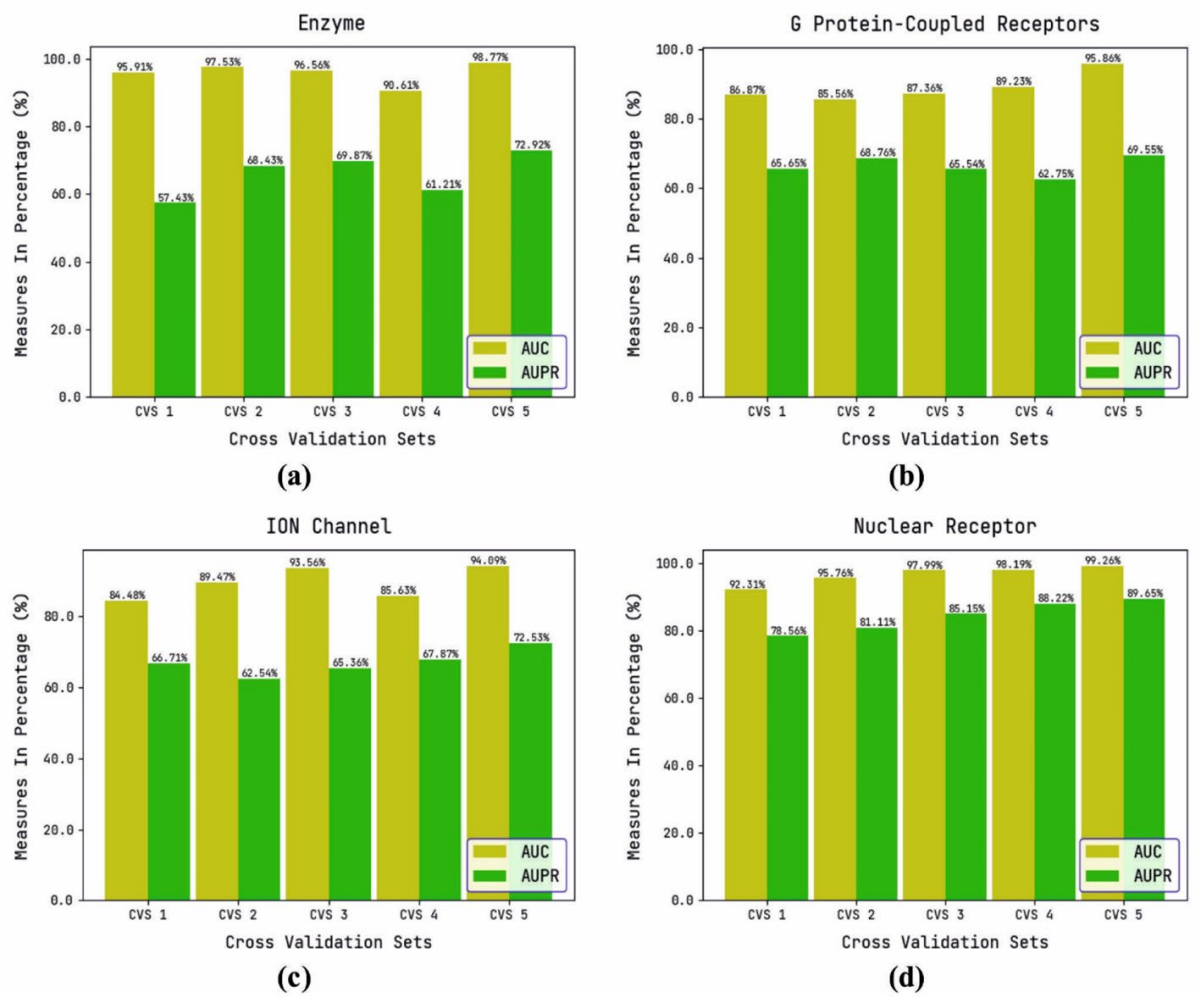
AUC and AUPR analysis of DTIP-WINDGRU model on various CV_SEEDs values. **a** Enzyme dataset, **b** G Protein-Coupled Receptors dataset, **c** ION Channel dataset, **d** Nuclear Receptor dataset, displaying AUC and AUPR performance across five cross-validation sets

Table 3 and Fig. 4 report detailed AUC and AUPR outcomes of the DTIP-WINDGRU model on the test datasets applied. The experimental results revealed that the DTIP-WINDGRU model has accomplished effective values of AUC and AUPR under distinct seed values for instance, with enzyme dataset and CV_SEED value of 3201, the DTIP-WINDGRU model has provided AUC and AUPR of 95.91%, and 57.43% respectively. In GPCR dataset and CV_SEED value of 3201, the DTIP-WDOGRU model has gained AUC and AUPR of 86.87%, and 65.65% respectively. Moreover, with ION Channel dataset and CV_SEED value of 3201, the DTIP-WDOGRU model has accomplished AUC and AUPR of 84.48%, and 66.71% respectively. Furthermore, with Nuclear Receptor dataset and CV_SEED value of 3201, the DTIP-WDOGRU model has provided AUC and AUPR of 92.31%, and 78.56% respectively.

**Table 3:** AUC and AUPR on Different CV_SEEDs Values of Applied Dataset

Enzyme dataset			GPCR dataset		
CV_SEED	AUC	AUPR	CV_SEED	AUC	AUPR
3201	95.91	57.43	3201	86.87	65.65
2033	97.53	68.43	2033	85.56	68.76
5179	96.56	69.87	5179	87.36	65.54
2931	90.61	61.21	2931	89.23	62.75
9117	98.77	72.92	9117	95.86	69.55
*ION channel dataset*			*Nuclear receptor dataset*		
3201	84.48	66.71	3201	92.31	78.56
2033	89.47	62.54	2033	95.76	81.11
5179	93.56	65.36	5179	97.99	85.15
2931	85.63	67.87	2931	98.19	88.22
9117	94.09	72.53	9117	99.26	89.65

A detailed comparative result analysis of the DTIP-WINDGRU model with recent models in terms of AUC on four test datasets is provided in Table 4.

**Table 4:** AUC analysis of DTIP-WINDGRU technique with existing methods under four datasets

Methods	Enzyme	GPCR	ION Channel	Nuclear receptor
UDTPP (Lan et al. 2016)	86.00	87.60	77.50	80.00
Bi-gram PSSM (Rajpura and Ngom, 2018)	94.80	88.90	87.20	86.90
Nearest Neighbour (Rajpura and Ngom, 2018)	89.80	88.90	85.20	82.00
IFB Model (Rajpura and Ngom, 2018)	84.50	81.20	73.10	83.00
KBMF2 K (Rajpura and Ngom, 2018)	83.20	85.70	79.90	82.40
DBSI (Rajpura and Ngom, 2018)	80.60	80.30	80.30	75.90
ColdDTA (Fang et al. 2023)	92.00	90.50	88.00	91.50
Drug Pocket Identification (Zhang et al., 2025)	85.2	89.7	83.5	88.1
DTIP-WINDGRU (Proposed)	98.77	95.86	94.09	99.26

Table 4 shows the comparative AUC results of the DTIP-WINDGRU model with existing models on enzyme and GPCR datasets. The results indicated that the DTIP-WINDGRU model has gained effectual results with maximum AUC on both datasets. The enzyme dataset, using DTIP-WINDGRU model has offered higher AUC of 98.77% whereas the UDTPP, Bi-gram PSSM, Nearest Neighbor, IFB, KBMF2 K, DBSI, ColdDTA [30] and Drug Pocket Identification models [33] have obtained lower AUC Of 86%, 94.80%, 89.80%, 84.50%, 83.20%, 80.60%, 92% and 85.2% respectively. The GPCR dataset utilizing DTIP-WINDGRU technique has accessible higher AUC of 95.86% whereas the UDTPP, Bi-gram PSSM, Nearest Neighbor, IFB, KBMF2 K, DBSI, ColdDTA and Drug Pocket Identification methods have reached lower AUC Of 87.60%, 88.90%, 88.90%, 81.20%, 85.70%, 80.30%, 90.50% and 89.7% correspondingly.

The comparative AUC results of the DTIP-WINDGRU methodology with existing models on ION channel and nuclear receptor datasets indicate that the DTIP-WINDGRU model has gained effective results with higher AUC on both datasets. The ION channel dataset, utilizing DTIP-WINDGRU methodology has offered higher AUC of 94.09% whereas the UDTPP, Bi-gram PSSM, Nearest Neighbor, IFB, KBMF2 K, DBSI, ColdDTA and Drug Pocket Identification systems have gained lower AUC Of 77.50%, 87.20%, 85.20%, 73.10%, 79.90%, 80.30%, 88% and 83.5% respectively. DTIP-WINDGRU algorithm on Nuclear receptor dataset has offered higher AUC of 99.26% whereas the UDTPP, Bi-gram PSSM, Nearest Neighbor, IFB, KBMF2 K, DBSI, ColdDTA and Drug Pocket Identification techniques have obtained minimal AUC Of 80%, 86.90%, 82%, 83%, 82.40%, 75.90%, 91.50% and 88.1% correspondingly.

**Fig. 5:**
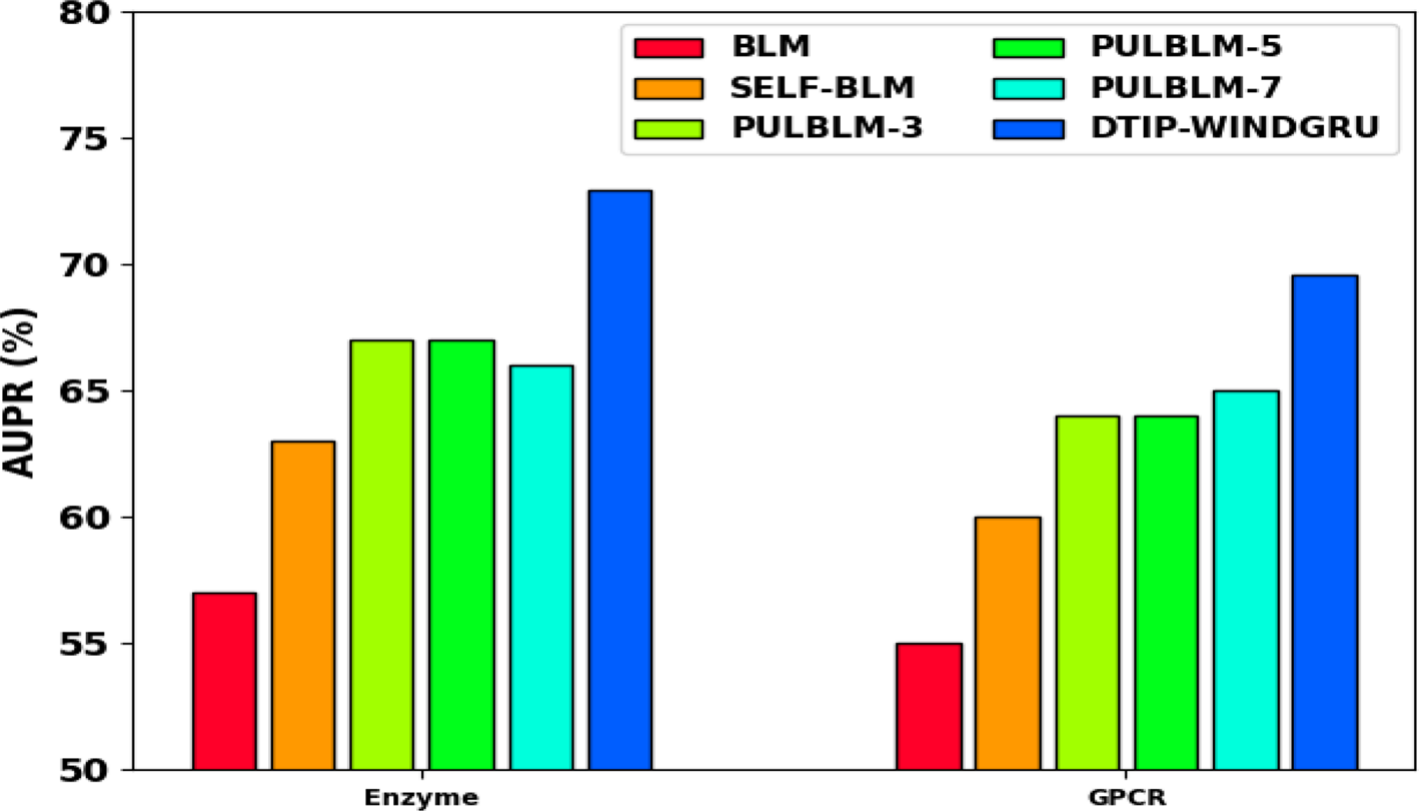
AUPR analysis of DTIP-WINDGRU model on Enzyme and GPCR datasets

A brief comparative result analysis of the DTIP-WINDGRU technique with recent methods in terms of AUPR on four test datasets is provided in Table 5 [27–29]. Figure 5 examines the comparative AUPR results of the DTIP-WINDGRU approach with existing models on enzyme and GPCR datasets. The results show that the DTIP-WINDGRU model has gained effective results with maximum AUPR on both datasets. The DTIP-WINDGRU system has offered higher AUPR of 72.92% on enzyme dataset whereas the BLM, SELF-BLM, PULBLM-3, PULBLM-5, and PULBLM-7 techniques have obtained lower AUPR of 57%, 63%, 67%, and 66% correspondingly. In addition, on GPCR dataset, the DTIP-WINDGRU approach has offered higher AUPR of 69.55% whereas the BLM, SELF-BLM, PULBLM-3, PULBLM-5, and PULBLM-7 models have obtained lower AUPR of 55%, 60%, 64%, 64%, and 65% correspondingly.

**Table 5:** AUPR analysis of DTIP-WINDGRU technique with existing methods under four datasets

Methods	Enzyme	GPCR	ION Channel	Nuclear Receptor
BLM (Haddadi and Keyvanpour, 2018)	57.00	55.00	47.00	42.00
SELF-BLM (Keyvanpour, 2018)	63.00	60.00	51.00	45.00
PULBLM-3 (Keyvanpour, 2018)	67.00	64.00	60.00	58.00
PULBLM-5 (Lan et al. 2016)	67.00	64.00	61.00	59.00
PULBLM-7 (Haddadi and Keyvanpour, 2018)	66.00	65.00	63.00	59.00
DTIP-WINDGRU (Proposed)	72.92	69.55	72.53	89.65

**Fig. 6:**
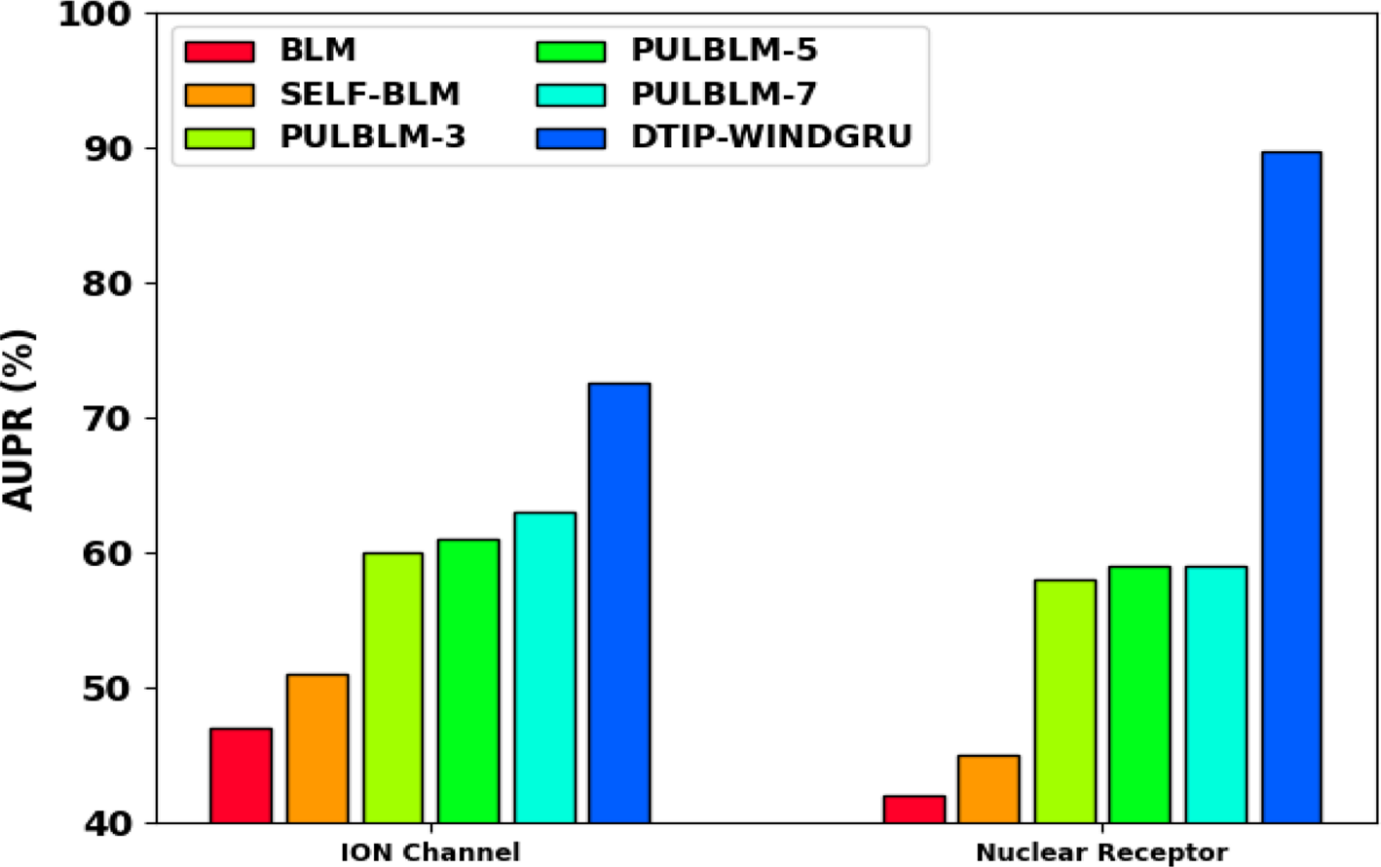
AUPR analysis of DTIP-WINDGRU model on ION channel and nuclear receptor datasets

Figure 6 shows the comparative AUPR results of the DTIP-WINDGRU algorithm with existing models on ION channel and nuclear receptor datasets. The results designated that the DTIP-WINDGRU technique has gained effectual results with maximal AUPR on both datasets. DTIP-WINDGRU model has offered higher AUPR of 72.53% on ION channel dataset whereas the BLM, SELF-BLM, PULBLM-3, PULBLM-5, and PULBLM-7 models have obtained decreased AUPR Of 47%, 51%, 60%, 61%, and 63% correspondingly. Eventually, on Nuclear receptor dataset, the DTIP-WINDGRU approach has offered higher AUPR of 89.65% whereas the BLM, SELF-BLM, PULBLM-3, PULBLM-5, and PULBLM-7 models have obtained lower AUPR of 42%, 45%, 58%, 59%, and 59% correspondingly.

After examining the comprehensive experimental analysis, it is concluded that the DTIP-WINDGRU model has accomplished maximum DTI predictive results over the other methods.

Table 6 shows a set of newer interactions for the proposed model DTIP-WINDGRU for each of the four datasets. These newer interactions were identified in publically available databases, proving the model’s efficacy in predicting undiscovered interactions from the available data. These interactions demonstrate the validity of our suggested model and demonstrate its applicability to the repurposing of currently prescribed medications for a wide range of unidentified disorders. It may also be used to real-world situations. For instance, in the case of SARS-CoV-2, it may be possible to find therapeutic compounds using drug repurposing from several heterogeneous sources. The use of computational approaches shortens the time and effort needed to find novel medications in wet laboratories. Our technique can advance the area of personalized medicine by selecting medications that are likely to be beneficial for certain individuals based on their genetic profiles and target protein interactions. Our approach predicts interactions between recently manufactured substances and target proteins, which can speed up the drug development process. This facilitates more effective drug candidates for pharmaceutical corporations, in order to evaluate the accuracy of the prediction, it is also advised to use already-approved medications for a condition that have been experimentally validated and further enhanced. It can also be used to identify interactions relating to uncommon disorders, where there may be a dearth of experimental data. The importance of our method in advancing drug discovery, streamlining drug development pipelines, and enhancing patient outcomes in the healthcare and biomedical fields is highlighted by these real-world applications. Finally, due to the use of deep learning techniques, our methodology requires vast amounts of data. However, the resulting new interaction data may be empirically validated to determine the success rate in the actual world.

**Table 6:** Results of proposed method on gold standard drug-target interaction dataset

Enzyme dataset				GPCR dataset			
Drug ID	Target ID	Inter. Conf	Database	Drug ID	Target ID	Inter. Conf	Database
D00097	hsa5743	0.9888	D and M	D00283	hsa1131	0.8028	D
D00569	hsa5742	0.9522	D	D00283	hsa1132	0.7999	D
D00418	hsa5742	0.9422	D	D00283	hsa1133	0.7592	D
D00448	hsa5743	0.9020	D and C	D00528	hsa1128	0.7023	M
D00448	hsa5742	0.8985	D and C	D00437	hsa1128	0.6819	M
D02561	hsa1565	0.7007	M	D00528	hsa1129	0.6541	M
D00512	hsa1565	0.6607	M	D00726	hsa1129	0.6346	M
D00279	hsa1565	0.6481	M	D00437	hsa1129	0.6263	M
D00043	hsa1636	0.6071	M	D01603	hsa154	0.6065	D
D00279	hsa1557	0.6037	M	D00283	hsa1814	0.6015	D, C, and M
*ION Channel Dataset*				*Nuclear Receptor Dataset*			
D00499	hsa1139	0.7066	D	D00554	hsa2100	0.7089	K
D03365	hsa1137	0.6518	D and C	D00312	hsa2100	0.8185	K
D03365	hsa1138	0.7025	D	D00067	hsa2100	0.6084	K
D03365	hsa1139	0.8053	D	D00898	hsa2100	0.6270	K
D02207	hsa1145	0.7448	K	D00962	hsa2100	0.8584	K
D02173	hsa1145	0.6091	D	D00443	hsa5241	0.6477	D
D00611	hsa1145	0.8291	K	D00443	hsa367	0.8252	D
D02356	hsa1134	0.6507	M	D00586	hsa2099	0.6068	M
D03742	hsa1137	0.8011	D	D00951	hsa2099	0.7647	D
D02356	hsa57053	0.6019	M	D00954	hsa367	0.8625	D

In order to validate the effectiveness of the WDO module, we conducted ablation experiments where we compared the performance of our DTIP-WINDGRU model with and without the WDO optimization represented in Table 7. The results of these experiments are as follows:

- *Baseline Model (Without WDO):* We trained the DTIP-GRU model using standard grid search and random search methods for hyperparameter tuning. The performance metrics (AUPR and AUROC) were recorded for the ION channel, Nuclear receptor, Enzyme, and GPCR datasets.
- *Enhanced Model (With WDO):* We then trained the DTIP-WINDGRU model using the Wind Driven Optimization algorithm for hyperparameter tuning. The same performance metrics were recorded for a fair comparison.

**Table 7:** Performance evaluation of DTIP-GRU model on gold standard drug-target interaction dataset

Dataset	AUPR (without WDO) (%)	AUPR (with WDO) (%)	AUROC (without WDO) (%)	AUROC (with WDO) (%)
ION channel	65.32	72.53	78.45	83.67
Nuclear receptor	82.14	89.65	88.23	92.15
Enzyme	70.12	76.84	82.67	87.45
GPCR	75.89	81.47	85.32	89.72

**Fig. 7:**
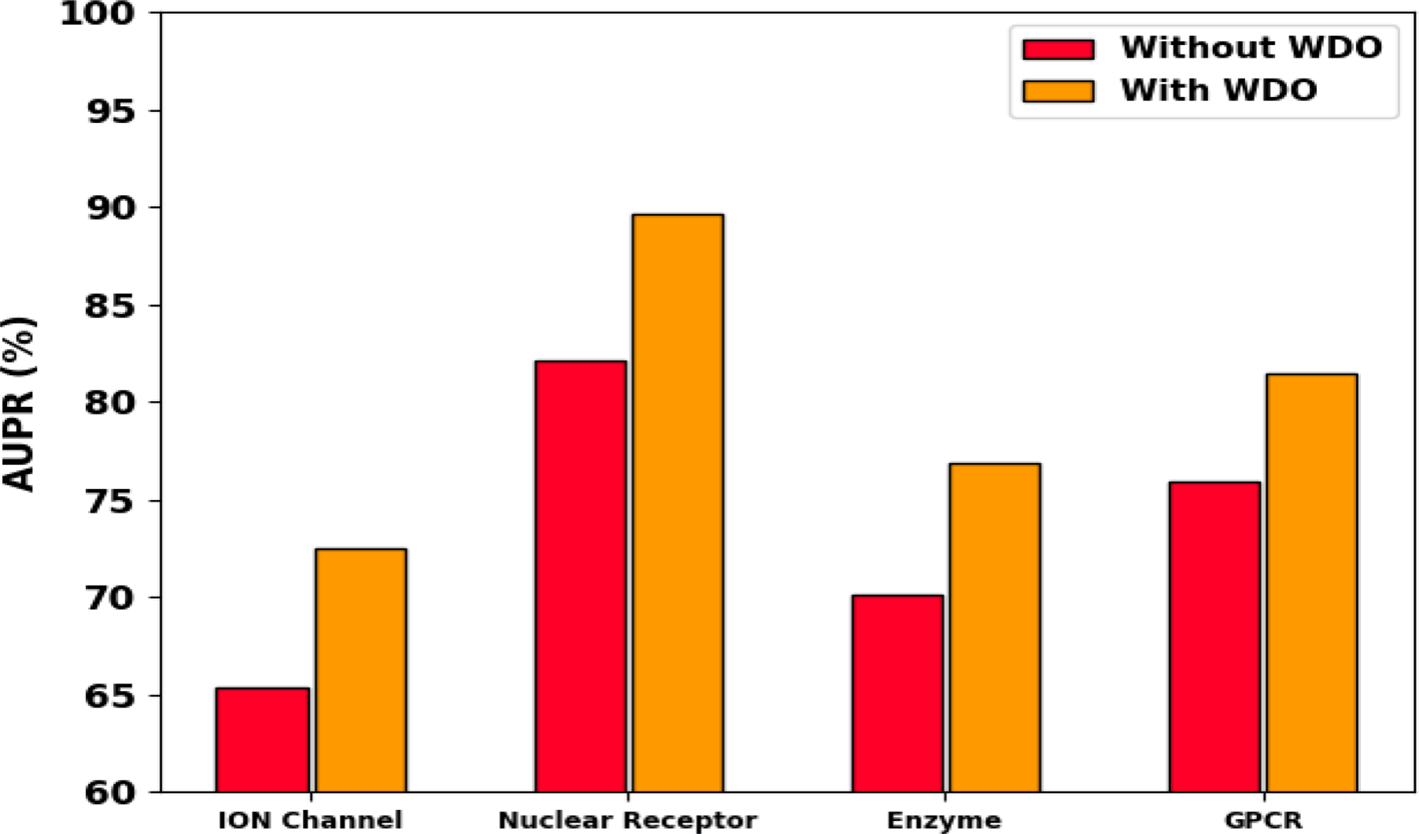
Ablation results of AUPR analysis with and without WDO in all four datasets

**Fig. 8:**
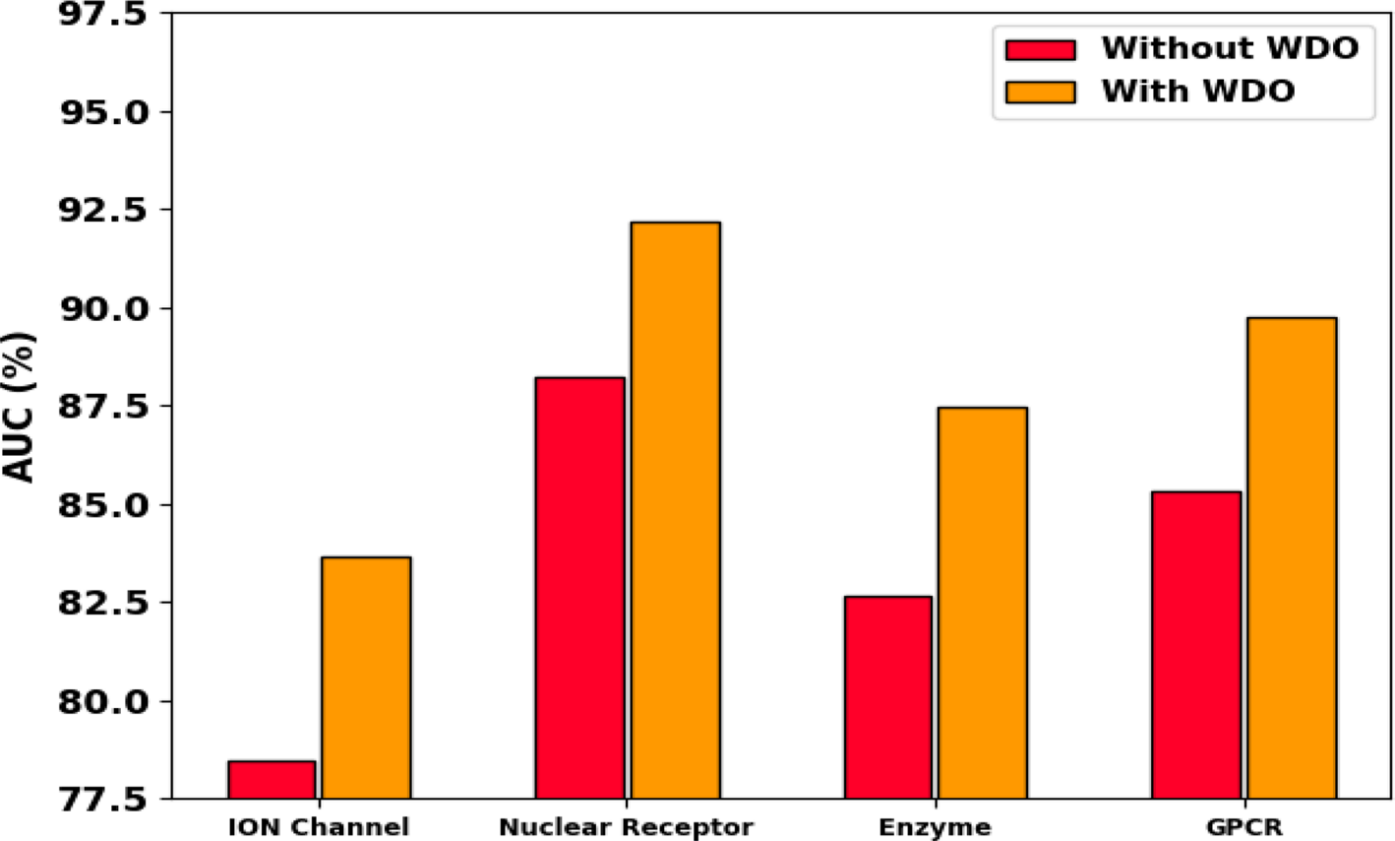
Ablation results of AUC analysis with and without WDO in all four datasets

The ablation experiment results in Figs. 7 and 8 clearly demonstrate the effectiveness of WDO in improving the model’s performance. The DTIP-WINDGRU model, with WDO, consistently achieved higher AUPR and AUROC scores compared to the baseline model without WDO. The incorporation of Wind Driven Optimization in our model significantly enhances its performance by efficiently exploring the hyperparameter space, leading to optimal settings that improve predictive accuracy. The results of the ablation experiments provide strong evidence of the effectiveness of the WDO module in the DTIP-WINDGRU model.

## Conclusion

This paper introduces a new model called DTIP-WINDGRU that integrates many methodologies and algorithms in order to improve the accuracy and effectiveness of DTIP. D-D and T-T interactions are used for weight initialization, GRU for prediction and hyperparameter optimization using the WDO technique. This is an important development since it enables the model to anticipate both known and prospective novel interactions, which is key for the development of new drugs. An important innovation is the use of the WDO method for hyperparameter optimization. Machine learning models must be tuned for optimal performance, and WDO provides a practical method for locating the best hyperparameters, improving the prediction outcomes. The proposed model has been experimentally validated, which elevates it to be useful for academics and professionals working in the pharmaceutical and biomedical industries. Real-world applications such as facilitating more effective drug candidates for pharmaceutical corporations and identifying interactions related to uncommon disorders highlights the significance of our proposed method. Additionally, developing personalized medicine by choosing medications that are likely to be beneficial for certain individuals, emphasizes the importance of our approach in advancing drug discovery, streamlining the drug development pipelines, and improving patient outcomes in the healthcare and biomedical fields. Our future work in drug discovery advancements include investigating hybrid DL models, which might produce even more precise DTIP predictions by combining several DL architectures or integrating DL with other computational techniques.

## Data Availability

The data that support the findings of this study are available from the corresponding author, upon reasonable request.

## Abbreviations

DTI: Drug target interactions
CI: Computational intelligence
ML: Machine learning
DL: Deep learning
GRU: Gated recurrent unit
D-D: Drug-to-drug
T-T: Target-to-target
DTIP-WINDGRU: Drug-target interaction prediction with wind-enhanced gated recurrent unit
WDO: Wind driven optimization
DTA: Drug-target binding affinity
QSAR: Quantitative structure–activity relationship
FDA: Food and drug administration
DeepDTA: Deep drug-target affinity prediction
WideDTA: Wide and deep learning for drug-target affinity prediction
GNNs: Graph neural networks
GraphDTA: Graph-based drug-target affinity prediction
MSGNN-DTA: Multi-scale graph neural network for drug-target affinity prediction
NHGNN-DTA: Node-adaptive hybrid graph neural network for drug-target affinity prediction
PSSM: Position specific scoring matrix
LM: Legendre moment
DeepLSTM: Deep Long Short-Term Memory
SPCA: Sparse Principal Component Analysis
DDR: Drug-target interaction prediction
SARS-CoV-2: Severe acute respiratory syndrome Coronavirus 2
MT-DTI: Multi-task drug-target interaction prediction
COPD: Chronic obstructive pulmonary disease
XGBoost: EXtreme gradient boosting
DNN-DTIs: Deep neural networks for drug-target interactions
CNN: Convolutional neural network
KG-DTI: Knowledge graph based DTI
AD: Alzheimer’s disease
GCN-DTI: Graph convolutional network DTI
DPPs: Drug-Protein Pairs
PU: Positive unlabeled
SVM: Support vector machine
PUDT: Positive unlabeled drug targets
PUL: Positive-unlabeled learning
BLM: Bipartite local model
GPCR: G-Protein Coupled Receptors
RNN: Recurrent neural networks
LSTM: Long short-term memory
FF: Fitness function
AUC: Area under the curve
AUPR: Area under the precision-recall curve
UDTPP: Unsupervised drug-target prediction with probabilistic pathway
IFB: Iterative feature boosting
KBMF2 K: Kernelized Bayesian matrix factorization with side information
DBSI: Drug-target binding site identification
ColdDTA: Cold-start drug-target affinity prediction
SELF-BLM: Self-boosting bipartite local model
PULBLM-3: Positive-unlabeled learning bipartite local model with 3 layers
PULBLM-5: Positive-unlabeled learning bipartite local model with 5 layers
PULBLM-7: Positive-unlabeled learning bipartite local model with 7 layers

## References

[1] Öztürk H, Özgür A, Ozkirimli E. DeepDTA: deep drug-target binding affinity prediction. Bioinformatics. 2018;34(17):i821–9. 30423097 10.1093/bioinformatics/bty593 PMC6129291

[2] Öztürk H, Ozkirimli E, Özgür A. WideDTA: prediction of drug-target binding affinity.2019. arXiv preprint arXiv:1902.04166. 10.1093/bioinformatics/bty593 PMC6129291 30423097

[3] Nguyen T, Le H, Quinn TP, Nguyen T, Le TD, Venkatesh S. GraphDTA: predicting drug-target binding affinity with graph neural networks. Bioinformatics. 2021;37(8):1140–7. 33119053 10.1093/bioinformatics/btaa921

[4] Wang S, Song X, Zhang Y, Zhang K, Liu Y, Ren C, Pang S. MSGNN-DTA: multi-scale topological feature fusion based on graph neural networks for drug-target binding affinity prediction. Int J Mol Sci. 2023;24(9):8326. 37176031 10.3390/ijms24098326 PMC10179712

[5] He H, Chen G, Chen CY. Integrating sequence and graph information for enhanced drug-target affinity prediction. Sci Chin Inform Sci. 2024;67(2):129101.

[6] Yousefi N, Yazdani-Jahromi M, Tayebi A, Kolanthai E, Neal CJ, Banerjee T, Gosai A, Balasubramanian G, Seal S, Ozmen GO. BindingSite-AugmentedDTA: enabling a next-generation pipeline for interpretable prediction models in drug repurposing. Brief Bioinform. 2023;24(3):bbad136. 37096593 10.1093/bib/bbad136 PMC10199763

[7] He H, Chen G, Chen CY. NHGNN-DTA: a node-adaptive hybrid graph neural network for interpretable drug-target binding affinity prediction. Bioinformatics. 2023;39(6):355. 10.1093/bioinformatics/btad355 PMC10287904 37252835

[8] Chen R, Liu X, Jin S, Lin J, Liu J. Machine learning for drug-target interaction prediction. Molecules. 2018;23(9):2208. 30200333 10.3390/molecules23092208 PMC6225477

[9] Wang YB, You ZH, Yang S, Yi HC, Chen ZH, Zheng K. A deep learning-based method for drug-target interaction prediction based on long short-term memory neural network. BMC Med Inform Decis Mak. 2020;20:1–9. 32183788 10.1186/s12911-020-1052-0 PMC7079345

[10] Lee I, Keum J, Nam H. DeepConv-DTI: prediction of drug-target interactions via deep learning with convolution on protein sequences. PLoS Comput Biol. 2019;15(6): e1007129. 31199797 10.1371/journal.pcbi.1007129 PMC6594651

[11] Bagherian M, Sabeti E, Wang K, Sartor MA, Nikolovska-Coleska Z, Najarian K. Machine learning approaches and databases for prediction of drug-target interaction: a survey paper. Brief Bioinform. 2021;22(1):247–69. 31950972 10.1093/bib/bbz157 PMC7820849

[12] Abbasi K, Razzaghi P, Poso A, Ghanbari-Ara S, Masoudi-Nejad A. Deep learning in drug target interaction prediction: current and future perspectives. Curr Med Chem. 2021;28(11):2100–13. 32895036 10.2174/0929867327666200907141016

[13] Jung YS, Kim Y, Cho YR. Comparative analysis of network-based approaches and machine learning algorithms for predicting drug-target interactions. Methods. 2022;198:19–31. 34737033 10.1016/j.ymeth.2021.10.007

[14] Feng Q, Dueva E, Cherkasov A, Ester M. Padme: A deep learning-based framework for drug-target interaction prediction. 2018. arXiv preprint arXiv:1807.09741.

[15] Olayan RS, Ashoor H, Bajic VB. DDR: efficient computational method to predict drug-target interactions using graph mining and machine learning approaches. Bioinformatics. 2018;34(7):1164–73. 29186331 10.1093/bioinformatics/btx731 PMC5998943

[16] Wang L, You ZH, Chen X, Xia SX, Liu F, Yan X, Zhou Y, Song KJ. A computational-based method for predicting drug-target interactions by using stacked autoencoder deep neural network. J Comput Biol. 2018;25(3):361–73. 28891684 10.1089/cmb.2017.0135

[17] Beck BR, Shin B, Choi Y, Park S, Kang K. Predicting commercially available antiviral drugs that may act on the novel coronavirus (SARS-CoV-2) through a drug-target interaction deep learning model. Comput Struct Biotechnol J. 2020;18:784–90. 32280433 10.1016/j.csbj.2020.03.025 PMC7118541

[18] Zhao T, Hu Y, Valsdottir LR, Zang T, Peng J. Identifying drug-target interactions based on graph convolutional network and deep neural network. Brief Bioinform. 2021;22(2):2141–50. 32367110 10.1093/bib/bbaa044

[19] Chen C, Shi H, Jiang Z, Salhi A, Chen R, Cui X, Yu B. DNN-DTIs: improved drug-target interactions prediction using XGBoost feature selection and deep neural network. Comput Biol Med. 2021;136: 104676. 34375902 10.1016/j.compbiomed.2021.104676

[20] Zhang P, Wei Z, Che C, Jin B. DeepMGT-DTI: transformer network incorporating multilayer graph information for drug-target interaction prediction. Comput Biol Med. 2022;142: 105214. 35030496 10.1016/j.compbiomed.2022.105214

[21] Wang S, Du Z, Ding M, Rodriguez-Paton A, Song T. KG-DTI: a knowledge graph based deep learning method for drug-target interaction predictions and Alzheimer’s disease drug repositions. Appl Intell. 2022;52(1):846–57.

[22] Rajpura HR, Ngom A. Drug target interaction predictions using PU-Leaming under different experimental setting for four formulations namely known drug target pair prediction, drug prediction, target prediction and unknown drug target pair prediction. In 2018 IEEE conference on computational intelligence in bioinformatics and computational biology (CIBCB). 2018 ; 1–7.

[23] Lan W, Wang J, Li M, Liu J, Li Y, Wu FX, Pan Y. Predicting drug-target interaction using positive-unlabeled learning. Neurocomputing. 2016;206:50–7.

[24] Haddadi F, Keyvanpour MR. PULBLM: A computational positive-unlabeled learning method for drug-target interactions prediction. In Proceedings of the 10th international conference on information and knowledge technology (IKT 2019), 2019; 31.

[25] Zhang YG, Tang J, He ZY, Tan J, Li C. A novel displacement prediction method using gated recurrent unit model with time series analysis in the Erdaohe landslide. Nat Hazards. 2021;105:783–813.

[26] http://web.kuicr.kyoto-u.ac.jp/supp/yoshi/drugtarget/

[27] Xu C, Shen J, Du X, Zhang F. An intrusion detection system using a deep neural network with gated recurrent units. IEEE Access. 2018;6:48697–707.

[28] Wang W, Liang S, Yu M, Liu D, Zhang H, Wang X, Zhou Y. GCHN-DTI: predicting drug-target interactions by graph convolution on heterogeneous networks. Methods. 2022;206:101–7. 36058415 10.1016/j.ymeth.2022.08.016

[29] Zhang Z, Chen L, Zhong F, Wang D, Jiang J, Zhang S, Jiang H, Zheng M, Li X. Graph neural network approaches for drug-target interactions. Curr Opin Struct Biol. 2022;73: 102327. 35074533 10.1016/j.sbi.2021.102327

[30] Fang K, Zhang Y, Du S, He J. ColdDTA: utilizing data augmentation and attention-based feature fusion for drug-target binding affinity prediction. Comput Biol Med. 2023;164: 107372. 37597410 10.1016/j.compbiomed.2023.107372

[31] Bayraktar Z, Komurcu M, Werner DH. Wind Driven Optimization (WDO): a novel nature-inspired optimization algorithm and its application to electromagnetics. In 2010 IEEE antennas and propagation society international symposium. 2010;1–4.

[32] Abdalla O, Rezk H, Ahmed EM. Wind driven optimization algorithm based global MPPT for PV system under non-uniform solar irradiance. Sol Energy. 2019;180:429–44.

[33] Zhang R, Chen Z, Li S, Lv H, Li J, Yang N, Dai S. Proteome-wide identification and comparison of drug pockets for discovering new drug indications and side effects. Molecules. 2025;30(2):260. 39860130 10.3390/molecules30020260 PMC11767986

